# Supplementary material for: Gait and Stability Analysis of People After Osteoporotic Spinal Fractures Treated with Minimally Invasive Surgery
Source: J Funct Morphol Kinesiol. 2025 Dec 17;10(4):481. doi: 10.3390/jfmk10040481 (PMC12734283; doi:10.3390/jfmk10040481)
Supplement: Supplementary file 1 [file jfmk-10-00481-s001.zip › jfmk-4015258-supplementary.pdf]

Supplementary Materials

Table S1. Norms for specific age ranges (Biodex, Shirley, NY), where: * - norms for studied people (13 people),** - norms for studied people (4 people).

Age range	17 – 35 years	36 – 53 years	54 – 71 years*	72 – 89 years**
FRT	0.7 – 2.1	0.7 – 3.1	0.9 – 3.7	2 - 4

Table S2. Mean (x) and standard deviation (SD) values of parameters describing the spatio-temporal gait structure in the patient group.

Spatio-temporal data	Patient Group		Norm	
	x	SD	x	SD
Stride rate [steps/min]	71.01	9.62	119.42	8.32
Gait cycle time [s]	1.71	0.41	1.01	0.31
Gait cycle length [m]	1.12	0.12	1.54	0.13
Single step length [m]	0.53	0.12	0.72	0.13
Mean gait velocity [m/s]	0.71	0.13	1.42	0.14

Table S3. Maximum and minimal values of angles in the stance and swing phases in the patient group and the normative data of the Vicon system – data in parentheses ().

	Hip Joint		Knee Joint		Ankle Joint	
Gait Cycle	Min [deg]	Max [deg]	Max [deg]	Max [deg]	Min [deg]	Min [deg]
Stance Phase (0 – 60)%	-3.7 ± 14.1 (-8.7 ± 5.8)	35.8 ± 12.4 (36.1 ± 12.4)	21.8 ± 13.3 (36.7 ± 5.2)		-3.39 ± 3.8 (-5.4 ± 2.1)	
Swing Phase (60 – 100)%		35.6 ± 14.3 (36.4 ± 5.4)		44.6 ± 16.8 (56.1 ± 3.7)		-2.3 ± 2.5 (-19.8 ± 6.9)

Table S4. Maximum and minimal values of muscle moments in the stance and swing phases in the patient group and the normative data of the Vicon system – data in parentheses ().

	Hip Joint		Knee Joint	Ankle Joint	
Gait Cycle	Max [Nm/kg]	Min [Nm/kg]	Max [Nm/kg]	Min [Nm/kg]	Max [Nm/kg]
Stance Phase (0 – 60)%	1.31 ± 0.37 (0.54 ± 0.25)	-0.45 ± 0.27 (-0.92 ± 0.19)	-0.33 ± 0.21 (0.56 ± 0.17)	0.004 ± 0.03 (-0.2 ± 0.06)	
Swing Phase (60 – 100)%					1.36 ± 0.29 (1.52 ± 0.16)

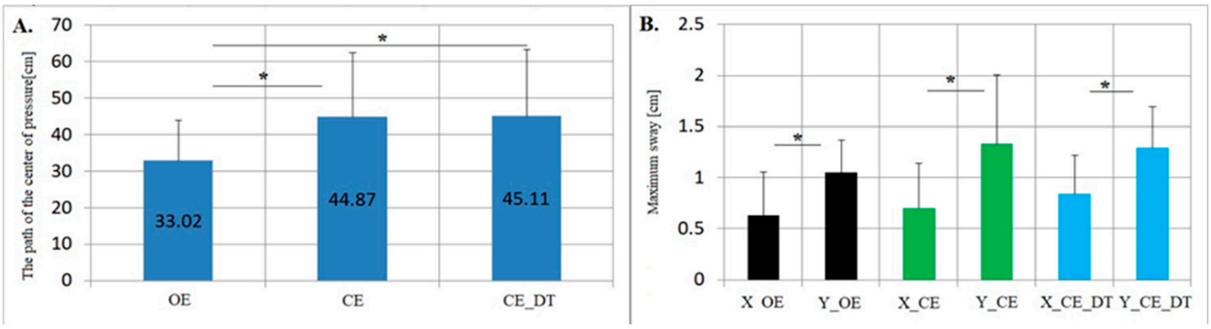

Figure S1. A. The path of the center of pressure of the feet on the ground when trying to stand both feet with eyes open (OE), eyes closed (CE) and with eyes closed and dual task (CE_DT), B. maximum sway in the sagittal(Y) and frontal(X) planes; where: * - statistically significant differences ($p < 0.05$).
